# Neglected Tropical Diseases and Other Infectious Diseases Affecting the Heart. The NET-Heart Project: Rationale and Design

**DOI:** 10.5334/gh.867

**Published:** 2020-09-01

**Authors:** Lucrecia M. Burgos, Juan Farina, Macarena Cousirat Liendro, Clara Saldarriaga, Alvaro Sosa Liprandi, Fernando Wyss, Ivan Mendoza, Adrian Baranchuk, On behalf of the Neglected Tropical Diseases and other Infectious Diseases affecting the Heart (NET-Heart project)

**Affiliations:** 1 Department of Heart Failure, Pulmonary Hypertension and Heart Transplant, Instituto Cardiovascular de Buenos Aires, Buenos Aires, AR; 2 Division of Cardiology, Clínica Olivos, Buenos Aires, AR; 3 Division of Cardiology, Hospital General de Agudos Carlos G. Durand, Buenos Aires, AR; 4 Department of Cardiology and Heart Failure Clinic, Cardiovascular Clinic Santa Maria, University of Antioquia, Medellín, CO; 5 Department of Cardiology, Sanatorio Güemes, Buenos Aires, AR; 6 Technology and Cardiovascular Service of Guatemala – Cardiosolutions, Guatemala City, GT; 7 Instituto de Medicina Tropical, Caracas, VE; 8 Division of Cardiology, Kingston Health Science Center, Queen’s University, Kingston, Ontario, CA

**Keywords:** neglected tropical diseases, cardiovascular disease, global health

## Abstract

**Introduction:**

Neglected tropical diseases (NTDs) are a group of infections that are prevalent in many of the tropical and sub-tropical developing countries where poverty is rampant. NTDs have remained largely unnoticed in the global health agenda. There is a substantial gap between the burden of disease for NTDs in cardiovascular diseases (CVD) and research devoted to the affected populations. We created a Latin-American initiative with emerging leaders (EL) from the Interamerican Society of Cardiology (IASC) with the objective to perform multiple systematic reviews of NTDs and other infectious diseases affecting the heart: The NET-Heart Project.

**Objective:**

To describe the rationale and design considerations of the NET-Heart project.

**Methods:**

The NET-Heart Project is a collaborative work of the IASC EL program. The main objective of the NET-Heart project is to systematically evaluate the available evidence of NTDs and other infectious diseases and their cardiovascular involvement. As a secondary objective, this initiative aims to offer recommendations and potential diagnostic and therapeutic algorithms that can aid the management of cardiovascular complications of these infectious diseases. After an expert discussion 17 initial infectious diseases were selected, for each disease we created one working group. The project was structured in different phases: Systematic review, brainstorming workshops, analysis and results, manuscript writing and recommendations and evaluation of clinical implications.

**Conclusion:**

The NET-Heart project is an innovative collaborative initiative created to assess burden and impact of NTDs and other infectious diseases in CVD. NTDs can no longer be ignored and must be prioritised on the health and research agenda. This project aims to review in depth the evidence regarding cardiac compromise of these serious conditions and to propose strategies to overcome barriers for efficient diagnosis and treatment of cardiovascular complications.

## Introduction

**Figure 1:**
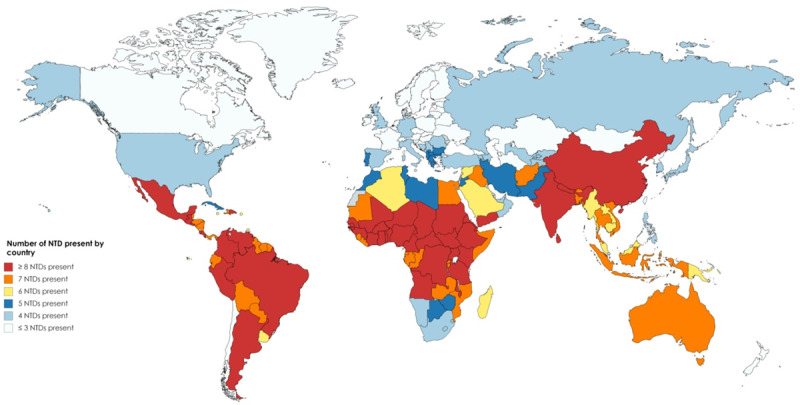
Number of NTDs present by country according to WHO estimates. Source: World Heart Organization [Internet]. Neglected tropical diseases; c2020 [cited 2020 July 04]. Available from: https://www.who.int/neglected_diseases/diseases/en/.

NTDs are a group of bacterial, parasitic, viral and fungal infections that are prevalent in many of the tropical and sub-tropical developing countries where poverty is rampant affecting more than one billion people [1]. By contrast, NTDs are quite rare in the more affluent countries of the developed world (Figure 1).

According to the last meeting of the Strategic and Technical Advisory Group for NTDs, the World Health Organization (WHO) has in its portfolio a diverse group of heterogeneous diseases, such as: Chagas disease, Buruli ulcer, Rabies, Soil-transmitted helminthiases, Trachoma, Schistosomiasis, Snakebite envenoming, Scabies and other ectoparasites, Taeniasis/Cysticercosis, Dengue and Chikungunya, Dracunculiasis (guinea-worm disease), Leishmaniasis, Leprosy (Hansen’s disease), Echinococcosis, Foodborne trematodiases, Human African trypanosomiasis (sleeping sickness), Onchocerciasis (river blindness), Lymphatic filariasis, Mycetoma, Yaws (Endemic treponematoses), chromoblastomycosis and other deep mycoses [1]. NTDs have been overlooked for decades but currently there is a growing awareness of their importance in the world’s lower middle-income countries (LMICs) [2].

NTDs cause a significant portion of disease. In the 2010 Global Burden of Disease Study, NTDs accounted for 26.06 million disability-adjusted life years (DALYs) [3]. Moreover, global attention tends to focus on killer diseases, though NTDs disable and disfigure more than they kill. DALYs due to NTDs constitute for 56% years lost due to disability (YLD) and for 44% years of life lost (YLL), as compared to 7% of YLD and 93% of YLL for malaria [4]. As a result, NTDs have remained largely forgotten in the global health agenda however more attention is currently being paid to this group of diseases. This last year WHO launched global consultations for a new Roadmap on NTDs [5]. A draft of the NTD Roadmap for 2021–2030 has just been published by the WHO and partners for further consultations [6]. The road map sets out global targets for 2030 and milestones to prevent, control, eliminate and eradicate a diverse set of 20 diseases and disease groups, while it also proposes strategies for attaining these targets over the next decade. Recently, during a collaboration of the World Heart Federation (WHF) and the IASC a road map on Chagas’ disease was created. It offers a comprehensive summary of current knowledge on prevention, diagnosis and management of the disease [7].

CVD is the leading cause of mortality worldwide [8]. More than 80% of these cases were in LMICs and nearly 40% of these are labelled as premature [9,10]. Despite the lower risk-factor burden in LMICs, the rates of major CVD and death were substantially higher in low-income countries than in high-income countries [11]. Traditional risk factors, such as smoking and obesity, may contribute to the increase in CVD in developing countries but they cannot provide the complete explanation for this phenomenon. An important component of the burden of CVD may be attributed to NTDs and other endemic diseases but little is known about the burden of CVD assigned to NTDs; adequate data is lacking to determine the true extent of CVD resulting from NTDs [12]. Almost one-half of this CVD burden is attributable to ischemic heart disease, more than one-third to cerebrovascular disease and the remainder to hypertensive and inflammatory causes as well as rheumatic heart disease. NTDs may account for a significant component of each of these CVD categories [13]. For all the above reasons, a global response to NTDs is warranted [14].

There is a substantial gap between the burden of disease for NTDs in CVDs and research devoted to the affected populations. Furthermore, considering the difficulties to access healthcare services that exist in many regions affected by NTDs, the usual recommendations may not be easy to implement so alternative strategies might be necessary. We created a Latin American initiative with the EL program from the IASC with the objective to perform multiple systematic reviews of NTDs and other infectious diseases affecting the heart: The NET-Heart Project.

## Objectives

The purpose of this paper is to describe the rationale and design considerations of the NET-Heart project.

## Methods

The NET-Heart project is a collaborative work of the IASC EL program.

**Figure 2:**
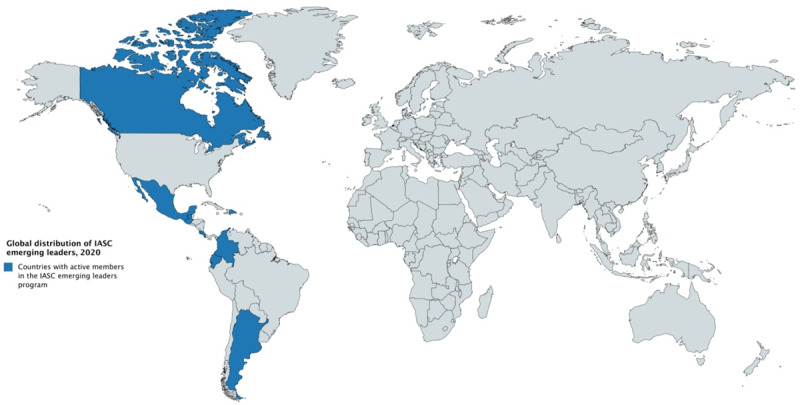
Global distribution of IASC emerging leaders, 2020. Source: Interamerican Society of Cardiology [Internet]. Emerging leaders program; c2020 [cited 2020 July 04]. Available from: http://www.siacardio.com/lideres-emergentes-siac/.

The EL program is comprised of a group of enthusiastic, talented and creative young cardiologists, residents, pre-grad students and fellows from different countries of north and south America (Figure 2).

The main objective of the NET-Heart project is to systematically evaluate the available evidence of NTDs and other infectious diseases and their cardiovascular involvement. As a secondary objective, this initiative aims to offer recommendations and potential diagnostic and therapeutic algorithms that can aid the management of cardiovascular complications of these infectious diseases.

After consulting experts in tropical diseases and cardiologists, we chose the infectious diseases that presented major impact on the cardiovascular system. We selected 17 infectious diseases: human immunodeficiency virus, dengue, zika, chikungunya, tuberculosis, African trypanosomiasis, malaria, Chagas, schistosomiasis, Lyme, cysticercosis, echinococcosis, fascioliasis, leishmaniasis, coronavirus disease 2019, rabies and toxoplasmosis.

For each disease we created one working group led by a principal investigator with a co-investigator, an expert on the infectious disease to be investigated, and a supervisor. Globally they had a director (AB) and coordinator (CS) (Table 1).

**Table 1:** Working groups and research topics.

Research topic	Principal investigator	Co-Principal investigator

**HIV & Heart**	Macarena Cousirat (Argentina)	Cristhian Ramírez (Colombia)
**Dengue & Heart**	Diego Araiza Garaygordobil (México)	Carlos Eduardo García (Guatemala)
**Zika & Heart**	Cristhian Emmanuel Scatularo (Argentina)	Oswaldo Andrés Ballesteros (Ecuador)
**Chikungunya & Heart**	Ana Laura Sauce Pérez (México)	Juan Ignacio Cotella (Argentina)
**Tuberculosis & Heart**	José Patricio López (Colombia)	Liliana Posada (México)
**African Trypanosomiasis & Heart**	Héctor Isaac Ortiz (Guatemala)	Juan Farina (Argentina)
**Malaria & Heart**	Shyla Gupta (Canada)	Naomi Gazendam (Canada)
**Chagas & Heart**	Andrés Felipe Miranda (Colombia)	Gonzalo Miranda (Argentina)
**Schistosomiasis & Heart**	Liliana Posada (México)	Luis Gerardo González (México)
**Lyme & Heart**	Cynthia Yeung (Canada)	Dennys Franco (México)
**Cysticercosis & Heart**	Carlos Eduardo Garcia (Guatemala)	Cristhian Emmanuel Scatularo (Argentina)
**Echinococcosis & Heart**	Oswaldo Andres Ballesteros (Ecuador)	Diego Araiza Garaygordobil (México)
**Fascioliasis & Heart**	Juan Ignacio Cotella (Argentina)	Ana Laura Sauce (México)
**Leishmaniasis & Heart**	Juan Farina (Argentina)	Carlos Eduardo Garcia (Guatemala)
**COVID-19 & Heart**	Cristhian Ramirez (Colombia)	Lucrecia Burgos (Argentina)
**Rabies & Heart**	Gonzalo Miranda (Argentina)	Jose Patricio Lopez (Colombia)
**Toxoplasmosis & Heart**	Zier Zhou (Canada)	Hector Isaac Ortiz (Guatemala)

The project was structured in four main phases.

- Systematic review: It includes studies evaluating the infectious disease and its cardiovascular involvement. We adhered to the Preferred Reporting Items for Systematic Reviews and Meta-Analyses (PRISMA) statement in conducting and reporting the systematic reviews [15].
- “Brainstorming” Workshops: Based on the systematic review research results, workshops were held with the experts to determine the focus and design of the study (comprehensive review and meta-analysis).
- Analysis and results.
- Manuscript writing and recommendations and evaluation of clinical implications.

All initial meetings and workshops were conducted through video conferences. Currently, the vast majority of the project’s working groups are starting phase 3.

## Conclusion

The NET-Heart project is an innovative collaborative initiative to assess burden and impact of NTDs and other infectious diseases in CVDs. Considering that the burden of traditional risk factors cannot fully explain the increase in CVDs in LMICs, and that there is lack of information about the contribution of NTDs to CVD, we aim to review in depth the evidence regarding cardiac compromise of these serious conditions. We also intend to propose strategies to overcome barriers for early diagnosis and efficient treatment of cardiovascular complications.
